# Hemangioblastoma in the Lung: Metastatic or Primary Lesions?

**DOI:** 10.1155/2014/468671

**Published:** 2014-12-14

**Authors:** Li Lu, Peter A. Drew, Anthony T. Yachnis

**Affiliations:** Department of Pathology, Immunology and Laboratory Medicine, University of Florida, Gainesville, FL 32610, USA

## Abstract

Hemangioblastoma primarily occurs in the CNS, most commonly in the posterior fossa. Extracranial locations are less common, and metastatic tumor involving the lung is exceedingly rare with only three cases previously reported. Two were autopsy studies in patients who died of complications of the CNS hemangioblastomas in 1943 and 1981, and the third was mentioned in a case report addendum providing follow-up information on hepatic hemangioblastoma in 1991. We report a case of a 48-year-old man who presented with multiple lung nodules treated by surgical excision. Pathological study revealed features classic for hemangioblastoma. The patient had a remote history of hemangioblastomas having been excised from the posterior fossa 7 and 20 years previously. This report details a fourth case of metastatic pulmonary hemangioblastoma. It is the first report on surgically resected hemangioblastomas from the lung of a living patient with histological and immunohistochemical characterization.

## 1. Introduction 

Hemangioblastoma is an uncommon tumor of the central nervous system (CNS), representing 1.5–2.5% of all intracranial neoplasms1. Within the CNS, the most common site is the posterior fossa where the tumor often forms a solitary nodule in the wall of a glial-lined cyst [1, 2]. About one-fourth of hemangioblastomas occur in patients with Von Hippel-Lindau disease (VHL); the remainders are sporadic. Although extremely rare, solid hemangioblastomas outside the CNS have been reported, involving peripheral nerve [3], retroperitoneum [4], soft tissue and bone [5–7], and visceral organs including the pancreas [8], adrenals [9, 10], liver [11], and lung [12, 13]. Most of the above cases of peripheral hemangioblastoma occurred in patients who also had CNS tumor with few exceptions [6]. Three cases of hemangioblastoma in the lung have been published in English literature. The first, reported by Abbott and Love in 1943 [12], concerned a 32-year-old man with recurrent right parasagittal frontal hemangioblastoma treated twice with surgical excision. At autopsy, multiple nodules involving all lobes of lung were identified and proven to be hemangioblastomas bearing the same histological features as the patient's previously removed brain tumors. The second case, reported in 1981 by Iannotti et al. [13], described a 52-year-old man with spinal hemangioblastoma persisting for 20 years and treated with multiple laminectomies. A lung nodule, again found at autopsy in the right lower lobe, was histologically consistent with hemangioblastoma. The third case was mentioned in an addendum of a case report by Rojiani et al. in 1991 [11] on a case of hepatic hemangioblastoma in a 39-year-old man with previously resected cerebellar and spinal hemangioblastomas who developed hepatic hemangioblastoma requiring liver transplant. Nodules of hemangioblastoma involving bilateral lungs were found six months after liver transplant. We now report a case of multiple hemangioblastomas in the lung in a patient with remote history of cerebellar hemangioblastoma.

## 2. Case Presentation 

We recently encountered a most unusual lung tumor case in our pulmonary pathology consultation service. The patient was a 48-year-old male who presented with history of a dry cough and occasional hemoptysis. A chest X-ray was performed, followed by a CT scan of the chest, which revealed a right lower lobe 2.5 cm lung mass and a 0.7 cm right upper lobe nodule. A PET/CT scan showed mild hypermetabolic uptake of the dominant 2.5 cm mass of the right lower lobe and no abnormalities of the neck, chest, abdomen, or pelvis.

**Figure 1:**
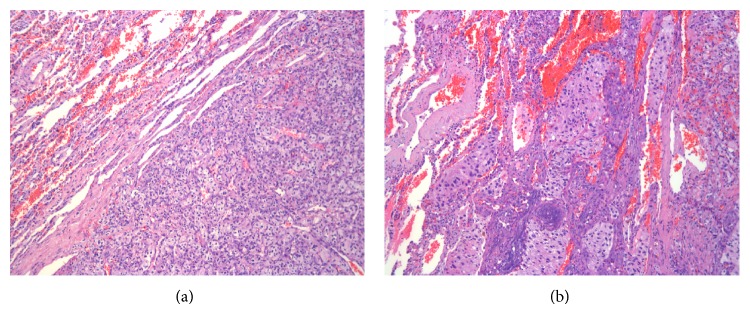
Well-demarcated but not encapsulated tumor with focal involvement of the adjacent alveolar air spaces (hematoxylin-eosin, original magnification ×100).

Pathologic material sent to our pulmonary pathology service for review consisted of sections from the right lower lobe lobectomy specimen containing the dominant tumor nodule and a wedge biopsy from the right upper lobe including the smaller lesion. Multiple mediastinal lymph nodes are also sampled and show no evidence of tumor. Microscopically, at low magnification, the tumors were unencapsulated (Figures 1(a) and 1(b)) but well circumscribed (Figure 1(a)) with a few solid tumor nests noted in the alveolar air spaces at the periphery of each lesion (Figure 1(b)).

**Figure 2:**
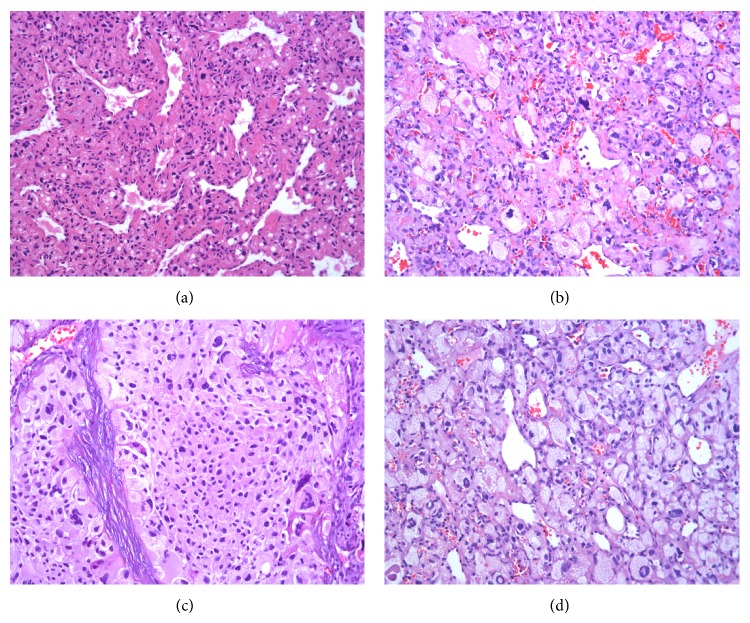
Variable histological features: prominent capillary network (a), pleomorphic nuclei (b and c), solid tumor nodules (c), and small cytoplasmic lipid vacuoles (d) (hematoxylin-eosin, original magnification ×200 (a)–(c), ×400 (d)).

Both tumors contained a prominent capillary network (Figures 2(a) and 2(b)) with intervening stromal cells exhibiting varying degrees of pleomorphism (Figures 2(b) and 2(c)). The cytoplasm of the stromal cells was eosinophilic to clear (Figures 2(b) and 2(c)) and many contained fine lipid vacuoles (Figure 2(d)). Occasional solid tumor lobules devoid of vasculature were also present, often associated with more cytological atypia (Figure 2(c)). Despite being highly vascular, the smaller (0.7 cm) tumor nodule showed primarily bland cytological features in the stromal cells as depicted in Figure 2(a).

Initial immunohistochemistry studies showed nonreactivity of the tumor for pancytokeratin, epithelial membrane antigen, synaptophysin, chromogranin, Melan-A, and CD10. These findings did not support the diagnosis of carcinoma, melanoma, or a neuroendocrine tumor. The lipid-containing tumor cells were negative for Melan A, a finding that is inconsistent with adrenal cortical neoplasm, although not exclusive. Additional immunohistochemistry studies revealed negative reactivity for the histiocytic markers CD68, CD1a, CD45, and lysozyme.

**Figure 3:**
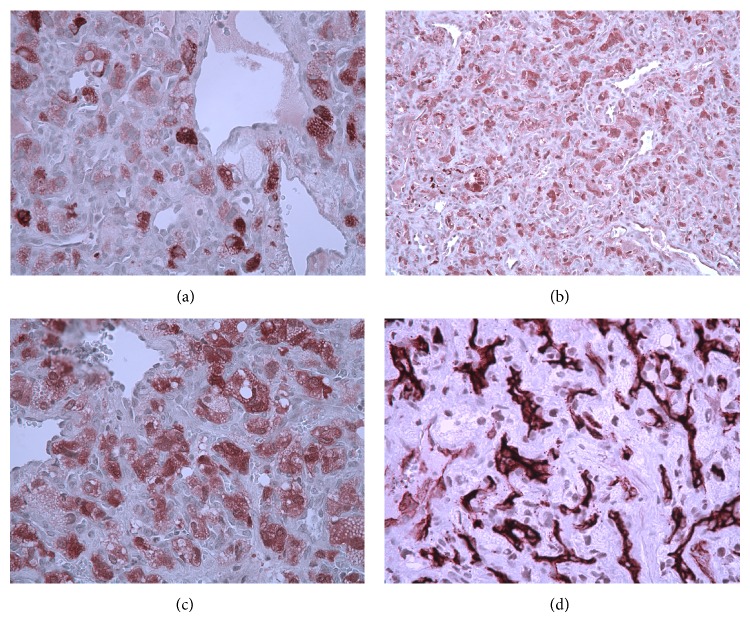
Positive immunoreactivity is characteristic of hemangioblastoma with the following markers: (a) inhibin-A, (b) neuron specific enolase (NSE), (c) S-100, and (d) CD34 (original magnifications for all images: ×400).

During work-up of this case via telecommunication with the hospitals where the patient was originally treated, we learned that the patient had two previous posterior brain surgeries, 20 years and 7 years prior to the discovery of the currently reported lung tumors. Based on this clinical information and histological features of the tumor described above further immunohistochemistry studies were undertaken to investigate the possibility of metastatic brain tumor especially hemangioblastoma. The tumor stromal cells were positive for inhibin-A, neuron specific enolase (NSE), S-100, and vimentin. Immunostains for CD34 were positive in the prominent capillary vasculature, but not in the stromal cells (Figure 3). Less than 5% of the tumor cells were reactive for Ki-67 (MIB-1). Immunostain for GFAP is negative. A summary of the immunohistochemical findings is shown in Table 1. Given the patient's prior history and the histological and immunohistochemical findings the lung masses were most likely metastatic hemangioblastoma to the lung.

At this point we received a copy of the surgical pathology report of the previous surgery performed seven years ago. The patient indeed had a “recurrent hemangioblastoma” removed from the posterior fossa per report.

**Figure 4:**
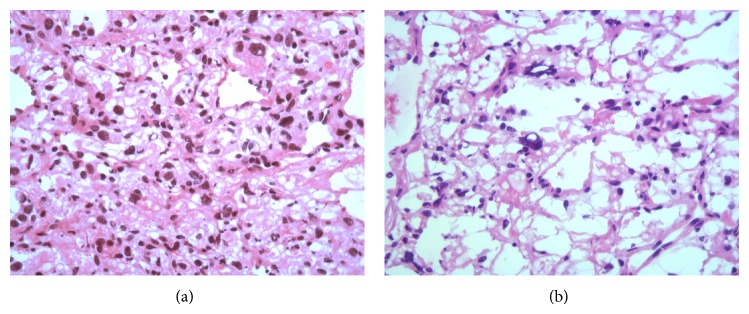
Histologic features on frozen sections of the lung mass from the right lower lobe: microcystic and vascular architecture and nuclear pleomorphism mimicking metastatic renal cell carcinoma (hematoxylin-eosin, original magnification ×400).

It bares mentioning that, on frozen section, the tumor appeared highly vascular with microcystic changes and tumor cells epithelioid with eosinophilic or vacuolated cytoplasm and pleomorphic nuclei mimicking renal cell carcinoma (Figures 4(a) and 4(b)).

## 3. Discussion 

All reported cases of pulmonary hemangioblastoma, including the current case, have presented as multiple discrete tumor nodules involving one or multiple lobes. Because such tumors are rare, hemangioblastoma is not usually included in the differential diagnosis of lung masses.

Such tumors are more likely to be interpreted as metastatic carcinomas, especially on frozen section. As shown in current case, microcystic changes and clear cell appearance on frozen sections may resemble renal cell carcinoma. It is therefore important to be aware of this rare entity and understand its unique clinical associations, key histological features, and immunohistochemical characteristics.

Most hemangioblastomas occur in the CNS, with a quarter of them arising in patients with Von Hippel-Lindau disease. Extracranial hemangioblastoma is less common but more often associated with VHL. Thus far, all cases of visceral organ hemangioblastoma in the literature are in patients either with familial VHL disease or with previous CNS tumor with surgical excision. The current patient did not have a family history of VHL at the time of surgery for the lung lesions. The history of recurrent cerebellar hemangioblastomas and subsequent multiple hemangioblastomas in the lung would certainly suggest the possibility of VHL.

Hemangioblastoma could be considered in the differential diagnosis of multiple well-demarcated, highly vascular tumor nodules, consisting of tumor cells with foamy cytoplasm containing fine lipid droplets and atypical nuclei but low mitotic activity and immunohistochemistry panels exclude carcinoma, melanoma, and histiocytic neoplasms. Other lipid-containing tumors such as adrenal cortical neoplasms, inhibin-positive sex cord stromal tumors, liposarcoma, and histiocytic lesions should be considered in the differential diagnosis.

Although the pathogenesis is incompletely understood, hemangioblastoma is generally believed to be a benign neoplasm originating in the CNS and associated with either germ-line or somatic mutations of the VHL gene on 3p25-26 [1, 2]. Whether the pulmonary tumors in this case represent “late metastases” from a primary cerebellar site or a new primary site remains problematic. The first reported case by Abbott and Love [12] was diagnosed as “malignant hemangioblastoma, grade III” according to the tumor classification in 1943. The tumor nodules in the lung and mediastinal lymph nodes discovered at autopsy were interpreted as metastasis in the report. The two patients reported by Iannotti et al., 1981 [13], and Rojiani et al., 1991 [11], had multiple operations prior to the discovery of lung tumors. Because of the short time interval between the surgeries and the discovery of pulmonary hemangioblastomas, the authors suggested postoperative spreading as the cause for lung metastasis. In fact, fatal dissemination of hemangioblastoma within CNS involving brainstem and spinal cord has been observed, suggesting the possibility of aggressive biologic behavior [14, 15]. We do not know if the primary hemangioblastoma has the capacity to invade blood vessel resulting in distant metastasis without surgical disruption of tissue integrity. However our case and previously reported cases indicate that rare examples of hemangioblastoma of the CNS may display visceral organ involvement and that such peripheral tumors may arise many years after initial diagnosis of CNS hemangioblastoma. Although unlikely, we cannot exclude the possibility that the pulmonary hemangioblastomas reported here could have arisen as primary lung tumors rather than metastases.

Several cases of metastasizing renal cell carcinoma have been reported in patient with a CNS hemangioblastoma [16] and therefore differential diagnosis of multiple metastatic tumors in the lung in this group of patients should include renal cell carcinoma. As shown in this case report (Figure 4), on frozen sections, the hemangioblastoma from the right lower lobe exhibits features mimicking clear cell renal cell carcinoma. Deferring the final diagnosis to permanent sections with immunohistochemistry would be the best choice in this situation.

In summary, we report the fourth documented case of hemangioblastoma in the lung, but, to our knowledge, this is the first report of the histological and immunohistochemical evaluation of such tumors being surgically excised from a living patient. Practicing surgical pathologists should be aware of the possibility of hemangioblastoma occurring in the lung. The clinical history of prior brain surgery, even in the remote past, should be sought in such cases. This case provides additional evidence for the metastatic potential of CNS hemangioblastoma.

## Figures and Tables

**Table 1:** Results of immunohistochemistry studies on pulmonary hemangioblastoma.

Antibodies	Immunohistochemistry results	Clone/dilution/company
Inhibin	Stromal cell positive	R1/prediluted/VMS^c^
NSE^a^	Stromal cell positive	Polyclonal/1 : 100/Zymed
S-100	Stromal cell positive	Polyclonal/1 : 900/DACO
Vimentin	Stromal cell positive	V9/prediluted/VMS^c^
Calretinin	Rare stromal cell positive	Polyclonal/1 : 25/Introgen
Pancytokeratin	Negative	AE1/3/PCK26/prediluted/VMS^c^
EMA^b^	Negative	E29/prediluted/Cell Marque
CD10	Negative	SP67/prediluted/VMS^c^
Melan A	Negative	MART-1/prediluted/VMS^c^
Synaptophysin	Negative	SP11/prediluted/VMS^c^
Chromogranin	Negative	Polyclonal/1 : 1600/DACO
GFAP	Negative	Polyclonal/1 : 1600/DACO
CD68	Negative	KP1/1 : 3600/DACO
Lysozyme	Negative	Polyclonal/1 : 3600/DACO
CD1a	Negative	EP3622/prediluted/VMS^c^
CD34	Endothelial cell positive	QBend10/prediluted/VMS^c^
Ki-67	Estimated 5% of interstitial tumor cells positive	MIB1/1 : 25/DACO

^a^NSE: neuron specific enolase

^b^EMA: embryonic membrane antigen

^c^VMS: Ventana Medical System Inc.

Note: CD34, lysozyme, calretinin, and NSE are done on small tumor nodule; the rest are done on larger tumor nodule.
